# Magnitude Processing in the Brain: An fMRI Study of Time, Space, and Numerosity as a Shared Cortical System

**DOI:** 10.3389/fnhum.2016.00500

**Published:** 2016-10-05

**Authors:** Kenny Skagerlund, Thomas Karlsson, Ulf Träff

**Affiliations:** ^1^Department of Behavioral Sciences and Learning, Linköping UniversityLinköping, Sweden; ^2^Linnaeus Centre HEAD, Linköping UniversityLinköping, Sweden

**Keywords:** number processing, time processing, spatial processing, magnitude processing, insula, intraparietal sulcus (IPS)

## Abstract

Continuous dimensions, such as time, space, and numerosity, have been suggested to be subserved by common neurocognitive mechanisms. Neuroimaging studies that have investigated either one or two dimensions simultaneously have consistently identified neural correlates in the parietal cortex of the brain. However, studies investigating the degree of neural overlap across several dimensions are inconclusive, and it remains an open question whether a potential overlap can be conceptualized as a neurocognitive magnitude processing system. The current functional magnetic resonance imaging study investigated the potential neurocognitive overlap across three dimensions. A sample of adults (*N* = 24) performed three different magnitude processing tasks: a temporal discrimination task, a number discrimination task, and a line length discrimination task. A conjunction analysis revealed several overlapping neural substrates across multiple magnitude dimensions, and we argue that these cortical nodes comprise a distributed magnitude processing system. Key components of this predominantly right-lateralized system include the intraparietal sulcus, insula, premotor cortex/SMA, and inferior frontal gyrus. Together with previous research highlighting intraparietal sulcus, our results suggest that the insula also is a core component of the magnitude processing system. We discuss the functional role of each of these components in the magnitude processing system and suggest that further research of this system may provide insight into the etiology of neurodevelopmental disorders where cognitive deficits in magnitude processing are manifest.

## Introduction

Time, space, and number are ubiquitous dimensions of human and animal lives, and independent research findings suggest that these magnitudes may be subserved by common neural correlates in the parietal cortex of the brain (Walsh, 2003; Kaufmann et al., 2008; Bueti and Walsh, 2009; Hayashi et al., 2013). It is argued that this shared magnitude system is an evolutionary product that is engaged during our everyday interactions with the external world. Our interactions require efficient coordination of these magnitudes, such as the integration of quantities, the amounts of visible berries in the immediate environment for example, and how distant they are in relation to our body. Moreover, reaching and grasping for these berries requires delicate integration of temporal and spatial information. Considerable research effort in various disciplines has now been directed toward understanding the nature of this presumably shared magnitude system, addressing questions such as whether there is a complete neurocognitive overlap between these different magnitudes or whether each magnitude dimension is processed by dissociated dimension-specific processes (e.g., Fabbri et al., 2012; Agrillo and Piffer, 2012; Hayashi et al., 2013; Vicario et al., 2013).

Research on numerical cognition has consistently pointed to the intraparietal sulcus (IPS) as a pivotal area implicated in representing abstract meaning of quantity (e.g., Ansari, 2008; Nieder and Dehaene, 2009; Kaufmann et al., 2011; Dastjerdi et al., 2013). It has been hypothesized that this quantity system forms the basis for the subsequent development of arithmetical abilities (e.g., Feigenson et al., 2004). Indeed, mounting evidence suggests that the ability to discriminate between sets of objects, taxing the so called *number sense*, is predictive of mathematical ability (Halberda et al., 2008; Piazza, 2010) and may play a role in developmental dyscalculia (Piazza et al., 2010; Mazzocco et al., 2011). A recent study by Harvey et al. (2013) using high field functional magnetic resonance imaging (fMRI) found a topographic representation of numerosity in the right IPS, where neural populations were sensitive to a preferred numerosity and tuning width. This is in line with research on macaque neurophysiology, which demonstrates that single neurons in the parietal cortex are tuned to specific numerosities (Nieder and Miller, 2004). This topographical representation of numerosity gives credence to the notion that humans share with other primates an evolutionary ancient number sense much like any other sensory system (Harvey et al., 2013). Further support for the notion that IPS has a central role in representing quantities comes from Dastjerdi et al. (2013) who studied three individuals diagnosed with epilepsy. These patients had intracranial implants surgically attached to the parietal cortex as part of the clinical treatment. The authors then used electrocortigography to record the intracranial recordings of neural signaling while administering a set of behavioral tasks. They found numerosity-specific activation in the IPS during an arithmetical experimental task and, intriguingly, even during casual social conversations where words signifying quantities were uttered (Dastjerdi et al., 2013). In addition, they also found neural signaling in the IPS when uttering words denoting general magnitude such as times and ordered events. These findings from the domain of numerical cognition unequivocally highlights the role of the IPS during numerical processing, but these findings are also suggestive that the functionality of the IPS may not be confined to numerosity alone, but rather that the IPS is a cortical hub involved in magnitude processing in general.

Processing of spatial information, such as of line length and mental rotation of objects, has also been linked to neurocognitive correlates in the IPS (Jordan et al., 2002; Fias et al., 2003; Milivojevic et al., 2009). Milivojevic et al. (2009) found a linear increase of activation in dorsal IPS with angular rotation on a mental rotation task, but they also found the same orientation dependent activation in pre-supplementary motor area (pSMA). One interpretation the authors made was that this fronto-parietal circuitry reflected a network devoted to visually guided action (Milivojevic et al., 2009). Congenial with this interpretation, Walsh (2003) proposed *A Theory of Magnitude* (ATOM), which is a model emphasizing the integrative role of visually guided action as the primary functional role of a shared magnitude system. By sharing neural substrates information across magnitudes can be incorporated, thereby supporting efficient coordination of these magnitudes that are relevant for action (Walsh, 2003; Bueti and Walsh, 2009).

Beside spatial and numerical information, converging evidence highlights the role of parietal cortex for temporal processing as well (Buhusi and Meck, 2005). Processing of temporal intervals is mainly connected to the prefrontal areas, such as the right inferior frontal gyrus (IFG) and SMA (Wiener et al., 2010) and right inferior parietal cortex (Lewis and Miall, 2003; Wittman, 2009; Bonato et al., 2012). Lewis and Miall (2006) proposed that the right prefrontal cortex and anterior insula together form a general-purpose system for cognitive time measurement. The role of the insula in time processing is consistently emphasized, especially during longer suprasecond intervals (Lewis and Miall, 2003). The functional role of the insula is currently debated, and it has been found to be involved during processing of sensory information of various modalities, such as during tactile stimulation and spatial discrimination (Pastor et al., 2006). Other researchers suggest that the insula, together with the anterior cingulate cortex (ACC), comprises a *salience network* (Menon and Uddin, 2010) that is responsible for the detection of environmentally salient events. The salience network regulates the deactivation of the default mode network (DMN) and the activation of the central executive network as a response to salient events that require attention (Menon and Uddin, 2010).

Hence, it is increasingly recognized that magnitude processing does not necessarily depend on any singular cortical location, but rather is heavily dependent upon complex neurocognitive circuitry and networks (e.g., Kosillo and Smith, 2010; Menon and Uddin, 2010; Hayashi et al., 2013). For example, Hayashi et al. (2013) showed that right intraparietal cortex (IPC) and right IFG are jointly activated by temporal and numerosity discrimination tasks. Notably, by using transcranial magnetic stimulation (TMS), they could identify the functional role of each of these cortical structures during these tasks. TMS of the right IFG impaired temporal discrimination, but did not impair temporal reproduction. Conversely, TMS to the IPC instead hampered temporal estimation. Thus, Hayashi et al. (2013) argued that right IFG is involved during a later stage in the magnitude processing chain, namely in the categorical decision stage, whereas the IPC is responsible for processing of numerosity and temporal magnitude representation in a previous stage.

Strong behavioral evidence for a shared magnitude system has been provided by experimental studies examining the interaction between magnitudes. A number of studies demonstrate a bidirectional interaction between space and number, that is, numbers influence performance on spatial tasks and space affects number processing (see Bueti and Walsh, 2009). Similar interactions have been found between number and time (e.g., Cappelletti et al., 2011). In contrast, Agrillo et al. (2010) failed to find an interaction between numerosity estimation and time estimation suggesting that time and number is processed by independent magnitude systems. Also space and time have demonstrated to exert reciprocal influence on each other (Ishihara et al., 2008; Fabbri et al., 2012; for methodological discussion see also Yates et al., 2012).

Additional behavioral evidence in support for a shared magnitude system comes from studies of atypical populations such as Developmental Dyscalculia (DD; Vicario et al., 2012; Skagerlund and Träff, 2014) and children with chromosome 22q11.2 deletion syndrome (Simon, 2008). Children with 22q11.2 deletions syndrome often have complex profile of cognitive dysfunctions, such as impaired visuospatial abilities, temporal abilities, and numerical abilities among others (Simon, 2008). This led Simon (2008) to propose that these individuals have a dysfunction in neural circuits subserving spatiotemporal processing, and that this spatiotemporal circuit provide the foundation for numerical representations as well. Simon (2008) hypothesizes that this neural dysfunction results in a spatiotemporal hypergranularity of the mental representations of these magnitudes, which subsequently impedes the functionality of higher order cognitive abilities dependent upon these magnitudes.

With respect to DD, Vicario et al. (2012) found that 8-year-olds with DD have weak time discrimination ability compared to controls, Moll et al. (2014) found that 9-year olds with DD were less accurate than controls, indicating that they have impaired time estimation for supra-second intervals specifically. Skagerlund and Träff (2014) showed that 10-years olds with DD displayed difficulties with time discrimination, but also with two spatial skills; spatial visualization (paper-folding) and mental rotation. In sum, even if behavioral studies examining time and spatial processing in children with DD are scarce, they suggest that impaired spatial and time processing might be two additional defining features of DD, and that time, space, and number may share the same neurocognitive resources and be part of a generalized magnitude processing system.

While the majority of prior neuroimaging studies have investigated magnitudes independently, a few studies have examined two dimensions together. Fias et al. (2003) compared the IPS activation of number magnitude comparisons with line lengths and angles comparisons. Cohen Kadosh et al. (2005) compared IPS activation while participants compared two digits on their numerical values, their height, and luminance. The posterior part of the left IPS was activated by all the comparison tasks in both studies and Cohen Kadosh et al. (2005) found also the same cluster was modulated by numerical distance effect, size distance effect, and luminance distance effect. Kaufmann et al. (2008) used an fMRI paradigm to study non-symbolic number decisions and spatial decisions. They found overlapping space and number regions in the right superior parietal lobe including the IPS. Pouthas et al. (2000) investigated time and numbers and showed that the activation in the right inferior parietal lobe was canceled out when the two conditions were subtracted from each other, suggesting that these dimensions are supported by similar cortical substrates. A recent study by Dormal et al. (2012) corroborates Pouthas et al. (2000) finding by showing that a duration task and numerosity task generated shared activation in a large right-lateralized fronto-parietal network, including the IPS and areas in the pre-central, middle, and superior frontal gyri. These findings provide converging evidence for the existence of a shared magnitude representation in the IPS.

In conclusion, even though fMRI studies investigating magnitude processing are scarce and produce mixed results they demonstrate, in line with the ATOM model, that the neural structures supporting the processing of time, space, and number is partially overlapping, but that the different magnitudes also draw on magnitude specific neural substrates. More specifically, according to available neuroimaging data the IPS appears to be the specific neural substrate for the general magnitude representation system. However, it is still unclear whether the locus of the general magnitude system involves both the left and right IPS, the right IPS specifically, and if also frontal, and possibly other areas are involved. Thus, the exact location of this shared magnitude system has not been clearly identified.

A limitation of prior research is that no neuroimaging study has directly investigated time, numbers and space within the same study. Using an fMRI paradigm, the aim of the current study is to contribute to our understanding of how the brain processes magnitude information across three dimensions – time, space, and number. We performed a whole brain voxel-wise analysis to explore the neural underpinnings of the processing of these dimensions, and we predicted that the IPS would be central hub of this system. In line with previous research we also hypothesized that the IFG would show conjoint activation across dimensions.

## Materials and Methods

### Participants

The initial sample included 29 right-handed, healthy adult students recruited from Linköping University. Due to excessive head motion (>4 mm along either axis), five participants were excluded from further analyses. The liberal motion threshold was because the paradigm was going to be used in children (with and without dyscalculia) and the threshold was the size of a voxel as in Park et al. (2014). We did not use motion parameters as covariates in the GLM. After motion correction and realignment we investigated motion parameters for each individual and looked at the results to examine potential motion artifacts. Thus, the final sample consisted of 24 individuals (14 females and 10 males) with a mean age of 24.33 (*SD* = 2.41). All participants had normal or corrected-to-normal vision, normal color vision, and no evidence or history of neurological illness or drug abuse. Written consent was obtained from each subject.

### Design and Experimental Tasks

**FIGURE 1:**
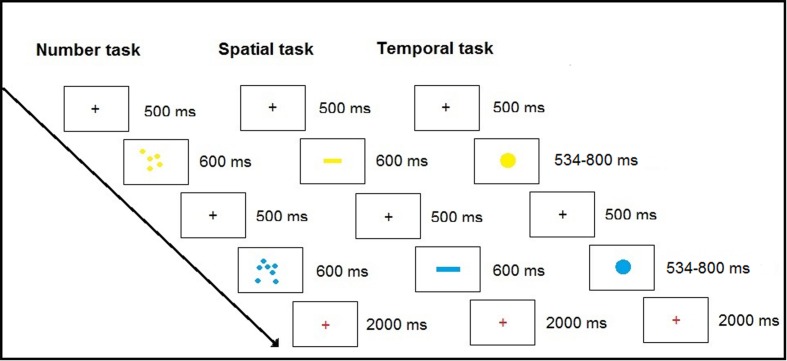
**Overview of the three magnitude processing tasks**.

The experiment consisted of three separate experimental tasks, each pertaining to one magnitude dimension (i.e., time, space, and number), and each of these tasks were accompanied with one control condition, in which the same stimuli were used and the same decision and motor processing were involved in all tasks. The tasks were administered using an alternating blocked design. Each experimental task was administered in eight separate blocks, each lasting 19 s. Control blocks were interspersed between experimental blocks, also in eight separate blocks of 19 s. An experimental block was immediately followed by a resting period of 12 s in order to get the hemodynamic signals back to baseline and provide a brief rest for the participants. After the resting period came to an end, a control block was introduced and subsequently followed by a rest period. During the rest periods, the participants simply fixated on a rest symbol. All experimental tasks as well as control tasks consisted of 32 trials, where both accuracy and RT was measured. After an experimental task was successfully completed in its entirety, an instruction screen was presented for 3 s which highlighted the fact that the next task was about to begin. The tasks were administered using SuperLab 4.5 (Cedrus Corporation, San Pedro, CA, USA). An overview of the fMRI paradigm can be found in **Figure 1** below. Each experimental task was accompanied with a control task, in which the same stimuli were used as in the corresponding experimental task. For example, after one block of the number discrimination task came to an end, the participants were administered the number control task. So in the context of the number control task, the subjects were presented with one array of yellow objects 600 ms, and then presented with a blue or yellow array of objects. The participants had to decide whether the arrays were of the same color. The task was structurally the same as the experimental task, but with the exception that they only had to pay attention to the color, and press one button if they matched, and another button if they did not. Participants responded by pressing one of two color-coded buttons on a Lumina response pad (Cedrus Corporation, San Pedro, CA, USA) for all tasks. In order to avoid task confusion, a cue indicating the upcoming task was presented briefly between blocks. The control task was identical for the other experimental tasks, but with the stimuli changed to match the congruent experimental task. The task order was the same for all participants. The participants started with the numerosity task plus control task, followed by the spatial task plus control task, and finally ended with the temporal task plus control task. A new cycle of tasks ensued, in the same task order, after a brief rest. Response times (RTs) were recorded during a response window that appeared immediately after the disappearance of the second comparison stimulus.

#### Number Discrimination

The numerical task required participants to discriminate between two sequentially presented arrays of blue and yellow dots (5–21 dots) after which they were asked to determine which array was more numerous. Each array was presented for 600 ms, and a blank screen separated them by 500 ms. This task was originally developed by Halberda et al. (2008) and only modified to present the arrays sequentially instead of simultaneously. Half of the trials were size controlled. Surface area was controlled for by having the cumulative surface area congruent with the number of dots in half the trials (i.e., cumulative area and number corresponded) and half the trials were incongruent. As in all tasks in this study, each trial ended with a response screen serving as a response window of 2000 ms across 32 trials. The ratios between the sets to be compared varied between 1:2, 2:3, and 3:4. The participants received no feedback regarding correctness of their response.

#### Line Length Discrimination

The spatial task was adapted from Fias et al. (2003) and Agrillo et al. (2013). Participants had to discriminate between two sequentially presented lines. Each trial consisted of one reference stimulus, in this case a yellow line, which was presented on the screen for 600 ms. A blank screen was presented for 500 ms after which the target stimulus appeared on the screen for 600 ms. The participants had to estimate which line was longer across 32 trials. Each trial ended with a response screen serving as a response window of 2000 ms. The lines varied in size between 300 × 30 pixels to 665 × 30 pixels and the ratios varied between 2:3, 3:4, and 4:5. The participants received no feedback regarding correctness of their response.

#### Time Discrimination

The temporal task involved making a judgment of which of two subsequently presented visual stimuli was presented the longest. A fixation cross was presented centrally on the screen for 500 ms, after which a yellow sphere (reference stimulus) was presented for 600 ms (stimuli duration varied between 534 and 800 ms), after which it disappeared and screen went blank for 500 ms. A blue sphere appeared on the screen and serving as a target stimulus and remained visible between 534 and 800 ms followed by a response screen visible for 2000 ms. Again, the ratios varied between 2:3, 3:4, and 4:5. The task consisted of 32 trials. The participants received no feedback regarding correctness of their response.

### fMRI Data Acquisition

The fMRI experiment was conducted at the Center for Medical Imaging and Visualization (CMIV), Linköping University, using a Phillips Ingenia 3T scanner. Images were acquired using a 3.0 T Phillips MRI scanner using a 32-channel head coil. Forty-nine 2.55 mm × 2.55 mm × 3.0 mm thick slices with in-plane resolution 3.0 mm isotropic, no gap, and ascending acquisition. Whole-brain functional scans were acquired with a T2^∗^-weighted echo planar imaging (EPI) pulse sequence (TR = 3000 ms, TE = 30 ms, flip = 90°) sensitive to blood oxygen level dependent (BOLD) contrasts. For each subject, a high-resolution structural scan was acquired with a T1-weighted pulse sequence (TR = 7.2 ms, TE = 2.8 ms, flip = 8°, slice thickness = 1.00 mm × 1.00 mm × 1.00 mm, number of slices = 170) after functional scans, to facilitate their localization and co-registration. Images were acquired in four runs in a single session, and each run comprised 170 volumes.

### fMRI Data Analysis

Preprocessing was performed using SPM8 (Wellcome Department of Cognitive Neurology, London, UK^1^) where functional images were motion corrected by realignment and the mean image was co-registered with the segmented anatomical image. Images were smoothed using a Gaussian kernel of 8 mm × 8 mm × 8 mm full width at half maximum (FWHM) and normalized into the default gray matter probability template in standard MNI (Montreal Neurological Institute) space. The general linear model (GLM) implemented in SPM8 was used for statistical analyses of BOLD images. A whole brain voxel-wise analysis was performed across subjects for each task in a two-stage analysis. For each subject, the signal from each experimental magnitude condition was contrasted with the paired control condition in the first-level analysis. A second-level random effects analysis for each magnitude contrast was then performed to obtain significant BOLD changes in the sample. For these analyses an uncorrected threshold of *p* < 0.001 was used. Moreover, to investigate the degree to which activations overlap across magnitude dimensions, a conjunction analysis with a familywise corrected threshold of *p* = 0.05 was performed over the contrasts [Number > Control] ∩ [Space > Control] ∩ [Time > Control]. A conjunction analysis takes into account the *t*-statistics, which are consistently high in the independent contrasts, and that become jointly significant in the conjunction analysis.

## Results

### Behavioral Results

A summary and overview of behavioral data can found in **Table 1**. RTs and accuracies were analyzed using two separate repeated measures ANOVAs for the three experimental conditions. ANOVA of mean RT between tasks showed a significant difference, *F*(2,46) = 157.44, *p* < 0.001, $\eta_{p}^{2}$ = 0.87. Three paired samples *t*-test were calculated between the three conditions to investigate this effect. After Bonferroni correction, the analyses showed no difference between the number and space condition (*p* = 0.227) but there was a difference between the number and time condition, *t*(23) = 14.04, *p* < 0.001, Cohen’s *d* = 3.00 and also between the space and time condition, *t*(23) = 14.45, *p* < 0.001, Cohen’s *d* = 3.07.

**Table 1:** Behavioral data of the magnitude processing tasks and control tasks.

	Response time (ms)		Accuracy (%)	
	RT	*SD*	Acc.	*SD*
Number discrimination	664	111	91.61	5.98
Number control task	644	93	97.26	3.03
Line length discrimination	633	114	96.25	4.28
Line length control task	624	109	98.74	2.03
Time discrimination	959	141	84.50	8.44
Time control task	597	73	98.39	2.21

The repeated measures ANOVA of accuracy showed a significant difference between the experimental conditions, *F*(2,46) = 21.27, *p* < 0.001, $\eta_{p}^{2}$ = 0.42. Pairwise comparisons were investigated after Bonferroni corrections and showed that the number and space condition differed significantly, *t*(23) = 3.52, *p* = 0.002, Cohen’s *d* = 0.6, that number and time also differed, *t*(23) = 3.36, *p* = 0.003, Cohen’s *d* = 0.83, and also that space and time differed significantly as well, *t*(23) = 6.15, *p* < 0.001, Cohen’s *d* = 1.50.

When comparing experimental conditions with their control conditions we found no difference in RTs in number vs. control (*p* = 0.251) or space vs. control (*p* = 0.428). The temporal task differed from its control, *t*(23) = 13.20, *p* < 0.001, *d* = 2.75. In terms of accuracies, number differed from its control, *t*(23) = 4.16, *p* < 0.001, *d* = 0.87, and space differed from its control, *t*(23) = 3.33, *p* = 0.006, *d* = 0.70, and time differed as well, *t*(23) = 7.77, *p* < 0.001, *d* = 1.62.

### Brain Imaging Results

#### Number Processing

**FIGURE 2:**
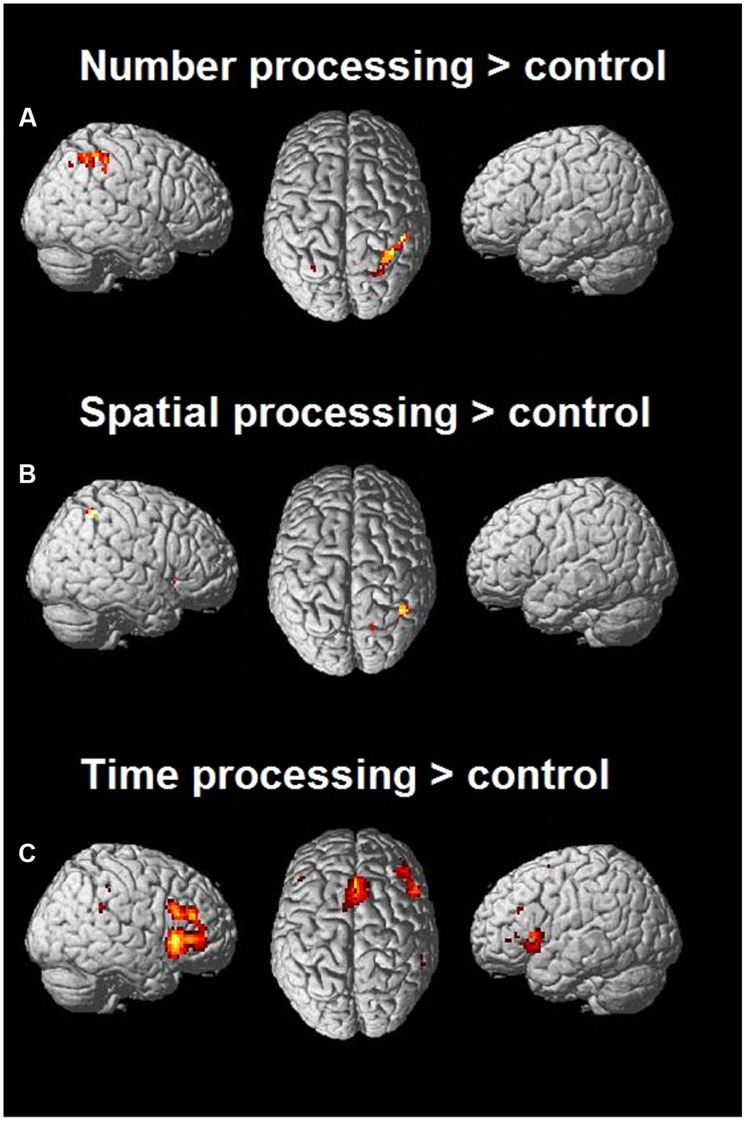
**Activation maps of each magnitude task contrasted with the control condition (*p* < 0.001 uncorrected)**. Significant activation during number processing can be seen in (A), and significant activation of spatial processing in (B), and time processing with significant activation can be seen in (C).

The aim of the first three analyses was to determine task related activation pertaining to each magnitude by contrasting them to each control condition. We used a probabilistic cytoarchitectonic map toolbox (Eickhoff et al., 2005) to report resulting activations of the different magnitude processing tasks. The activation patterns elicited in the number processing task could primarily be found in parietal cortices as expected (see **Table 2**), specifically in the posterior part of the IPS (hIP3) extending to surrounding parietal areas (see **Figure 2A**) in a predominantly right lateralized pattern. We also found involvement of the right insula.

**Table 2:** Clusters identified in the Number > Control contrast.

Anatomical region	MNI coordinates	BA	Cluster size	*p*	*Z*	Cohen’s *d*
Right superior occipital gyrus	27, -66, 46	7	52	<0.001	4.95	2.8
Right inferior parietal lobule	50, -36, 52	40	91	<0.001	4.91	2.31
Right intraparietal sulcus (hIP3)	34, -48, 48	7		<0. 001	4.82	2.26
Right superior parietal lobule	44, -43, 55	7		<0.001	4.81	2.25
Right insula	30, 26, -2	13	6	<0.001	4.87	2.73
Left intraparietal sulcus (hIP3)	-27, -61, 46	7	5	<0.001	4.58	2.48

*Value of cluster size indicates number of voxels. Coordinates indicate peak level activation*.

#### Spatial Processing

The line discrimination task showed similarly pronounced activation pattern in the right hemisphere as the number task when contrasted with the control condition (see **Table 3**; **Figure 2B**). The spatial task elicited signals in both the pars orbitalis and pars triangularis of the right IFG. The contrast also revealed activity pertaining to the right IPS, precuneus bilaterally, and the right insula.

**Table 3:** Clusters identified in the Space > Control contrast.

Anatomical region	MNI coordinates	BA	Cluster size	*p*	*Z*-score	Cohen’s *d*
Right superior occipital gyrus	27, -64, 31	7	114	<0.001	3.86	1.93


Right precuneus	19, -55, 25	7		<0.001	3.77	1.87


Left precuneus	-14, -69, 31	7	40	<0. 001	3.65	1.79


Right inferior frontal gyrus (PO^∗^)	32, 31, -5	47	24	<0.001	3.59	1.75


Right intraparietal sulcus (hIP3)	44, -51, 55	7	18	<0.001	3.40	1.63


Right superior parietal lobule	16, -66, 55	7	3	<0.001	3.37	1.62
Right inferior frontal gyrus (PT^∗∗^)	37, 28, 13	45	1	0.001	3.09	1.45
Right insula	42, 18, -5	13	1	0.001	3.09	1.45

*^∗^Pars orbitalis; ^∗∗^pars triangularis. Value of cluster size indicates number of voxels. Coordinates indicate peak level activation*.

#### Time Processing

Significant activations after contrasting the control condition could be located bilaterally in the insula, frontal eye-fields, and pars triangularis of the IFG. The right ACC was also involved during this task. Activations in the parietal cortex were mainly found in right SMG (see **Table 4**; **Figure 2C**).

**Table 4:** Clusters identified in the Time > Control contrast.

Anatomical region	MNI coordinates	BA	Cluster size	*p*	*Z*	Cohen’s *d*
Right insula	37, 28, 4	13	716	<0.001	5.59	3.45
Right inferior frontal gyrus (PT^∗^)	47, 41, -2	47		<0.001	4.54	2.45


Right frontal eye-fields	4, 21, 46	8	309	<0. 001	5.09	2.88


Left frontal eye-fields	-7, 5, 51	8		<0.001	3.53	1.78
Left insula	-30, 23, 1	13	181	<0.001	4.49	2.40
Left inferior frontal gyrus (PO^∗∗^)	-50, 15, 4	47		<0.001	3.77	1.87
Right posterior cingulate cortex	5, -18, 37	23	126	<0.001	3.53	1.77
Left inferior frontal gyrus (PT^∗^)	-45, 41, 1	47	2	<0.001	3.31	1.59
Right supramarginal gyrus	57, -36, 43	40	2	0.001	3.12	1.47

*^∗^Pars triangularis; ^∗∗^pars opercularis. Value of cluster size indicates number of voxels. Coordinates indicate peak level activation*.

#### Conjunction Analysis of Space, Time, and Number

**FIGURE 3:**
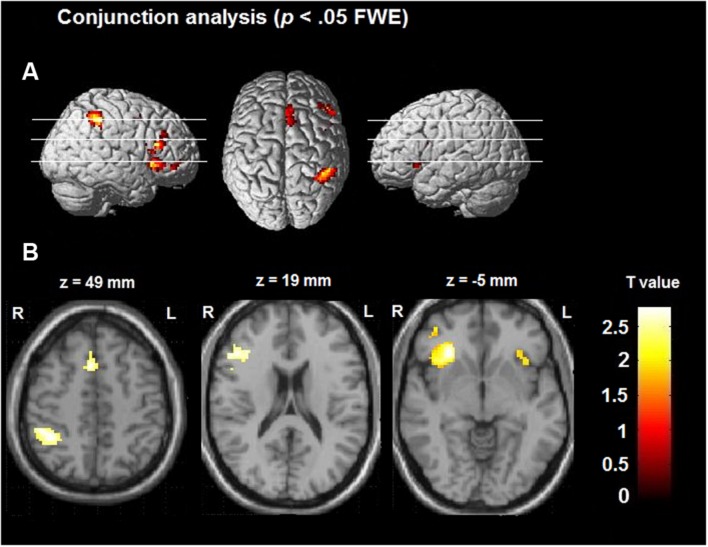
**Overlapping neural activations across all magnitude processing tasks including a rendition of the overlapping activations in (A) and slices indicating areas of interest in (B)**.

A conjunction analysis was performed over the three contrasts [Number > Control] ∩ [Space > Control] ∩ [Time > Control], which allows inferences to be made regarding significant and shared effects across all three magnitude dimensions. After FWE correction (<0.05), significant BOLD activations could be found primarily in the right hemisphere (see **Table 5**; **Figure 3**). The only cortical structures in the left hemisphere that contributed across all three magnitude tasks was the insula and the IFG. Significant activations in the frontal areas could be found in the right IFG, premotor cortex/SMA, DLPFC, and insula. More involvement of posterior regions could be traced to posterior cingulate cortex and the right IPS.

**Table 5:** Overlapping clusters identified in the conjunction analysis (FWE corrected < 0.05).

Anatomical region	MNI coordinates	BA	Cluster size	*p*	*Z*-score
Right inferior fontal gyrus (PO^∗^)	32, 28, -5	47	302	<0.001	7.35
Right insula	37, 23, 4	13		<0.001	5.82
Right middle frontal gyrus (DLPFC)	39, 33, 31	9		0.001	5.47
Right intraparietal sulcus (hIP2)	47, -41, 49	40	172	<0.001	5.68
Right supplementary motor area	6, 15, 52	6	119	<0.001	5.68
Right middle orbital gyrus	39, 51, -2	10	15	0.004	5.10
Left insula	-37, 21, -5	13	18	0.003	5.15
Left inferior frontal gyrus (PO^∗^)	-30, 28, -5	47		0.005	5.05
Right posterior cingulate cortex	1, -25, 31	23	2	0.026	4.67
Left inferior frontal gyrus (PO^∗∗^)	-45, 8, 22	44	1	0.034	4.61
Right inferior frontal gyrus (PO^∗∗^)	52, 13, 19	44	1	0.036	4.59
Right premotor cortex	44, 5, 52	6	1	0.047	4.52

*^∗^Pars orbitalis; ^∗∗^pars opercularis. Value of cluster size indicates number of voxels. Coordinates indicate peak level activation*.

Given the difference in reaction times between tasks, we wanted to rule out the possibility that the effects could be attributed to task difficulty. Therefore, we created two ROIs (right IPS and Insula) to see if there were any correlations between activation in these areas and RT. The results showed no significant correlations between RT and BOLD activation. Correlation coefficients in IPS in number contrast was *r* = -0.299, *p* = 0.165, in the spatial condition the correlation was, *r* = 0.20, *p* = 0.360, and in time it was *r* = -0.158, *p* = 0.471. In the insula the corresponding correlations were *r* = -0.24, *p* = 0.266; *r* = 0.245, *p* = 0.260; *r* = 0.154, *p* = 0.482. Thus, we could not establish a link between RT’s and activation, and hence the results do not indicate that difficulty was the driving factor behind the reported effects.

## Discussion

The aim of the present study was to shed light on the hypothesized existence of a generalized magnitude system and to investigate the neural substrates and potential overlap across dimensions. We hypothesized that the IPS and IFG would play a role in this magnitude processing network and that a conjunction analysis would reveal common activation patterns in these areas. To answer this research question, we utilized an fMRI paradigm consisting of three experimental tasks involving processing of space, time, and numerosity. The first step was to assess task-specific activation by contrasting each task with its designated control condition. A conjunction analysis was then performed and revealed a set of cortical loci involved across all tasks.

As predicted, the results revealed that the right IPS was involved in all three tasks and therefore gives credence to the notion that the IPS may play a constitutive role in the magnitude processing system and function as central hub responsible for the abstract representation of magnitude beyond numerosity alone. In addition, and as predicted, the conjunction analysis also revealed that the IFG was conjointly activated across tasks, which corroborates the findings by Hayashi et al. (2013). Their TMS experiment indicates that the right IFG plays a very specific role in the decision stage during magnitude processing. The tasks used in our study involve making comparisons and discrete decisions about two sequentially presented entities of magnitude, which may explain the cortical activation and functional role of the IFG in our study. This assertion is also reconcilable with Rusconi et al. (2011) who found that repetitive TMS to the IFG disrupted the *SNARC (Spatial Numerical Association of Response Codes;* Dehaene et al., 1993) *effect*. The SNARC effect is a phenomenon where individuals make faster left-sided responses to smaller numbers and faster right-sided responses to larger numbers when asked to estimate which of two simultaneously presented numerals is the largest. The disruption of this effect through TMS may indicate that the right IFG may play a key role in the spatial representation of numbers – the so called *mental number line*. The right IFG has also been implicated during spatial tasks and has been identified as a node in a ventral circuit for spatial attention (Corbetta and Shulman, 2002) and in the spatial representation of number (Doricchi et al., 2005). Rusconi et al. (2011) suggest that the right IFG and right frontal eye-fields are important areas involved during explicit magnitude comparisons, while also conceding that parietal circuits may also carry out spatial coding of numbers as well. We suggest that the right IFG play a specific role in spatially derived magnitude comparisons. This is in line with Wiener et al. (2010) and their meta-analysis of studies investigating temporal processing in the brain, where they found that the right IFG was involved in all temporal processing tasks irrespective of paradigm or temporal interval.

Another frontal area involved during all the magnitude processing tasks was the DLPFC, which is a region previously suggested to play a role in executive functions and working memory ability (Barbey et al., 2013). The contribution of the DLPFC while engaged in these tasks is not surprising given the sequentially presented stimuli and resultant demands on working memory. Each task requires that the participant holds a previously presented reference stimulus briefly in mind while focusing on a target stimulus. Although the working-memory demands are not high while carrying out the tasks, a quantum of working memory resources must still be engaged to make the magnitude comparisons.

The premotor cortex and SMA were other components involved during the magnitude processing tasks. Premotor cortex has connections with the parietal lobe, and these areas have been suggested to play a role in external sensory-guided actions and integration of different spatial reference frames (e.g., Kakei et al., 2001; McGuire and Sabes, 2011). The ATOM model emphasizes the integrative role of visually guided action as the primary functional role of a shared magnitude system (Bueti and Walsh, 2009), which may explain the coupling between magnitudes and motor actions subserved by the IPS and premotor cortex. Andres et al. (2008) demonstrated that digit magnitude influenced the way in which participants performed grip actions to digit stimuli. Closure of the grip action was initiated faster for smaller digits than for larger digits, and opening of the grip was faster for larger digits than for smaller digits. In addition, this influence of digit magnitude on grip aperture diminished as the distance between the hand and the object decreased, which led Andres et al. (2008) to argue that magnitude relates to actions primarily during the planning phase. Furthermore, Cisek and Kalaska (2005) utilized single-cell recordings of the premotor cortex in monkeys and found that when monkeys were presented with two options to reach for, the motor system initially represented both options. However, when cued about which object to reach for, the neural signature in the non-cued option was suppressed and the signature for the cued option was strengthened. The authors went on to argue that the premotor cortex represents possible action plans (Cisek and Kalaska, 2005). The idea that the premotor cortex represents possible action plans would explain the activity in the premotor cortex during all magnitude tasks in the present study as revealed by our conjunction analysis. A tentative interpretation of our findings is that the IPS represents amodal magnitude which is then projected via longitudinal fasciculus to anterior structures such as the premotor cortex and SMA to code for possible actions. Indeed, structural connections between anterior IPS and premotor cortex have been demonstrated using probabilistic cytoarchitectonic maps and resting-state connectivity analyses (Uddin et al., 2010). The authors also found that the anterior-most IPS was both structurally and functionally connected to the insula as well.

The notable involvement of the bilateral insulae in magnitude processing was a somewhat unexpected finding considering that this structure has not been widely reported in the literature on numerical or spatial cognition. One exception is the work by Pesenti et al. (2000) who reported specific activations in the right insula and IPS during processing of numerical stimuli. The right prefrontal cortex and anterior insula has been suggested to constitute a general-purpose system for cognitive time measurement (Lewis and Miall, 2006), so it was not surprising that the insula showed significant BOLD activation during the timing task of our study. However, our results indicate that the role of the insula may be generalized to encompass additional dimensions than being limited to processing of time alone. Corroborating this interpretation is the work by Nenadic et al. (2003); these authors reported increased activity in the insula during a frequency discrimination task as well as during an auditory time processing task. In addition, Ferrandez et al. (2003) reported conjoint activation during tasks requiring time discrimination and intensity discrimination. In the light of these findings, Kosillo and Smith (2010) suggested that the insula plays a role in sensory discrimination in general.

Although a limited number of previous studies and in particular the findings documented in this report highlight an important function for the insula in magnitude processing, the bulk of neuroimaging findings involving the insula have emphasized its role in detection of saliency. Menon and Uddin (2010) suggested the workings of a core salience network (see also Cole and Schneider, 2007; Dosenbach et al., 2007; Uddin, 2015). Involving the insula and the ACC, the function of the salience network is to identify stimuli from the continuous stream of sensory stimuli, and mark such stimuli for additional processing and initiate control signals (Menon and Uddin, 2010; Uddin, 2015). Extending this perspective, Craig (2009) proposed that salient interoceptive and environmental factors are coded moment-to-moment to represent a phenomenological “now,” the basis for self-awareness (for a critical dictum, however, see Philippi et al., 2012).

Clearly, our results depart from these notions on saliency and the workings of an alleged salience network in two ways. First, we did not find the often observed conjoint activation between the insula and the ACC. ACC activation typically involves processing of emotions (Klumpp et al., 2013), attentional control (Weissman et al., 2006), error monitoring (Klein et al., 2013), and effort-related processing (Engström et al., 2015). We believe that this lack of ACC involvement comes of little surprise, as the different tasks were carefully designed in terms of task difficulty and ratios to be compared.

Second, and more important to the objectives of this report, the insula was strongly activated in tasks tapping magnitude processing, rather than saliency. It could certainly be argued that these are highly similar concepts, in that saliency, similar to numerosity or duration, always is a prothetic dimension. However, the opposite may not always be true: larger numbers need not be more salient. In particular, in a context as the present experiment, it seems farfetched to conclude that seven dots on a display as compared to four dots invariably are more salient than five dots as compared to four dots. Rather than invoking saliency as an explanation for magnitude effects in the insula, our data suggest a direct role for the insula in magnitude processing. This property of the insula then could make it critical for the detection of saliency. Indeed, Sridharan et al. (2008) used Granger causal analysis to show that the insula predicted activity of the central executive network, which includes posterior parietal cortex and IPS, suggesting that activity in the insula projects information to parietal areas. Moreover, Uddin et al. (2010) showed structural connectivity between insula and anterior IPS. The authors suggested that IPS receives information from visual cortices and projects this information via the dorsal visual stream to anterior insula (Uddin et al., 2010). Hence, in terms of magnitude processing, the anterior insula may receive input from topographic representations of magnitude in the IPS (Harvey et al., 2013) and initially mark events, such as quantity or temporal units. The cardinality of magnitude may then be encoded and represented in the IPS, after which it is fed back to the insula and marked as a magnitude baseline with which subsequent stimuli can be contrasted and deemed as salient. The functional role of the insula may therefore be construed as involving both bottom–up detection and elongated activation to appropriate magnitude as salient units.

By administering three different tasks measuring different dimensions of magnitude, we find support for the existence of a generalized magnitude processing system. However, it should be noted that there is an ongoing debate about the specificity and purity of number processing. In terms of task purity of number discrimination tasks, it is difficult to disentangle the influence of confounding perceptual cues that correlate with numerosity (such as area density and occupancy) and may drive task performance (cf. Leibovich and Henik, 2013). More importantly, a recent review also calls into question the very existence of the number sense (see Leibovich et al., in press) and instead argues that number processing may be subserved by processing continuous magnitudes more generally. Our current findings could be interpreted as being in line with Leibovich et al. (in press), and future studies should focus on whether or not numerosity processing should be replaced with the notion of magnitude processing instead.

In sum, we have identified several overlapping neural substrates across multiple magnitude dimensions, and we argue that these cortical nodes comprise a distributed magnitude processing system congenial with the ATOM account (Walsh, 2003; Bueti and Walsh, 2009). Key components of this predominantly right-lateralized system include the IPS, insula, premotor cortex/SMA, and IFG. Moreover, this magnitude system can be understood using the framework of salience processing (Menon and Uddin, 2010). It should be noted, however, that the tasks used in our fMRI paradigm involve magnitude *discrimination* and not solely magnitude *representation*; as such, it is important to interpret the current findings in terms of these cognitively and behaviorally complex tasks. Thus, to disentangle the functional roles of the conjoint activations revealed by the conjunction analysis, additional analyses must be performed that enable inferences about directionality the information flow between the cortical regions in this system.

Another potential limitation is that the tasks differed with respect to reaction times and accuracies. Reaction times of the experimental tasks varied between 633 and 959 ms and accuracies ranged from 85 to 96%. Even if one is concerned about task difficulty, this would have posed a greater concern if we wanted to investigate and make inferences about unique contributions pertaining to each magnitude. Even comparing number and space, with a minor RT difference (664 vs. 633 ms), showed a tendency toward reaching significance (*p* = 0.076). One could arguably use RT as a covariate to control for these effects. However, imaging data and behavioral data are both dependent variables and thus they are both effects of magnitude processing and functional differences pertaining to magnitude. The behavioral data (i.e., RT/accuracy) does not cause the imaging data, and it would be unsound to covary the effects of behavioral data that may remove reliable hemodynamic effects pertaining to the processes under investigation (Henson, 2005).

Nevertheless, the primary goal of this study was to investigate the hypothesized magnitude system and contribute to our understanding of how the brain processes magnitude. Previous research using behavioral interference paradigms (e.g., Cappelletti et al., 2011; Fabbri et al., 2012) and behavioral tasks in general (Agrillo et al., 2013) have provided support for the notion of a shared magnitude processing system in line with ATOM. Neuroimaging studies employing two tasks have shown common activation patterns originating around the IPS (e.g., Fias et al., 2007; Kaufmann et al., 2008; Hayashi et al., 2013), and we extend those findings by utilizing three tasks pertaining to space, time, and number. Novel insights include the degree of overlap between several cortical areas, including premotor cortex and bilateral insula. Follow-up studies should focus on trying to understand the structure of this magnitude processing system and see if, as some researchers have suggested, mensuration is carried out by conversion of magnitudes to amodal ratios or proportions (e.g., Balci and Gallistel, 2006) and that the unifying principle in this shared magnitude system may indeed be proportion or ratio processing.

Future studies should clarify the interaction between the cortical areas of the magnitude processing system and take advantage of a systems neuroscience perspective and investigate functional and structural connectivity of the magnitude system in both typical and atypical populations. Additional insight can be gained from combining TMS and fMRI to elucidate the functional role of these nodes in the magnitude processing system (e.g., Hayashi et al., 2013). A developmental perspective (e.g., Kaufmann et al., 2008), allowing investigations across several age groups, can also provide fruitful knowledge about the development of the magnitude processing system throughout ontogeny. These approaches may yield insight into the etiology of disorders such as DD and 22q11.2 deletion syndrome, in which processing of magnitude has been found to be implicated.

## Conclusion

We have identified several overlapping neural substrates across multiple magnitude dimensions, and we argue that these cortical nodes comprise a distributed magnitude processing system congenial with the ATOM account (Walsh, 2003; Bueti and Walsh, 2009). Key components of this predominantly right-lateralized system include the IPS, insula, premotor cortex and IFG. We made tentative interpretations of the functional role of each of these components, where the right IPS is suggested to represent cardinal properties of magnitude which is then projected to the insula, involving salience detection and awareness of magnitude, and premotor cortex that codes magnitude for possible plans of action. The right IFG is involved in categorical decision-based representations of the magnitudes fed through the dorsal visual stream. The results suggest that the insula and the IPS are the core components of the magnitude processing system. We suggest that future research should delineate and verify the functional role of each of these components, which ultimately give insight into the etiology of neurodevelopmental disorders, such as DD and 22q11.2 deletion syndrome, where cognitive deficits in magnitude processing are manifest.

## Author Contributions

KS contributed by designing the study, collecting data, performing data analyses, and writing a draft of the manuscript. TK contributed to the design and the data analyses. UT contributed to the design of the study.

## Conflict of Interest Statement

The authors declare that the research was conducted in the absence of any commercial or financial relationships that could be construed as a potential conflict of interest.

## Footnotes

1 http://www.fil.ion.ucl.ac.uk
